# A Review of Machine Learning and AI Applications in Enhancing HACCP Systems for Ice Cream Manufacturing

**DOI:** 10.3390/foods15162815

**Published:** 2026-08-12

**Authors:** Juan Pablo Gaona Hernandez, Gbemileke Moses Olapade, Ha-Seong Cho, Hyun-Mo Jung, Myung-Hee Lee, Won-Young Lee

**Affiliations:** 1 School of Food Science and Technology, Kyungpook National University, Daegu 41566, Republic of Korea; 2 Division of High-Tech Agricultural Industry, Kyongbuk Science University, Chilgok 39913, Republic of Korea; 3 Research Institute of Tailored Food Technology, Kyungpook National University, Daegu 41566, Republic of Korea; 4 Department of Biomedical Technology, School of Convergence, Kyungpook National University, Daegu 41566, Republic of Korea

**Keywords:** artificial intelligence, food safety, HACCP, ice cream, machine learning

## Abstract

Hazard analysis and critical control point (HACCP) systems provide a preventive framework for food safety by implementing quality assurance plans, continuous monitoring, corrective actions, and risk mitigation strategies at critical control points throughout food processing, including dairy products such as ice cream. Artificial intelligence (AI) is increasingly transforming food safety management by enabling real-time monitoring, predictive analytics, and automated decision-making within food processing systems. This review critically examines the integration of AI technologies into HACCP systems for ice cream manufacturing, with an emphasis on improving hazard detection, process control, traceability, and the efficiency of corrective actions. The review evaluates the application of Internet of Things sensors, computer vision, and machine learning-based predictive monitoring systems across critical processing stages, including raw material reception, pasteurization, continuous freezing, and hardening/storage. Compared to conventional HACCP systems, AI-assisted technologies offer greater capabilities for anomaly detection, predictive maintenance, automated verification, and data-driven risk management. Nevertheless, their industrial implementation remains constrained by data quality limitations, infrastructure cost, cybersecurity risks, regulatory uncertainty, and limited model explainability. Accordingly, this review highlights key research gaps related to industrial scalability, validation under dynamic processing conditions, and the scarcity of ice cream-specific AI datasets. Finally, the review identifies future research directions and emerging opportunities for applying AI technologies in food processing and quality control systems, providing a framework for the evolution of intelligent HACCP systems in frozen dairy manufacturing.

## 1. Introduction

Hazard analysis and critical control point (HACCP) systems remain central to modern food safety management, providing a preventive framework for controlling biological, chemical, and physical hazards throughout food processing operations. In dairy and frozen dessert manufacturing, HACCP is particularly important for minimizing microbial contamination, maintaining product quality, and ensuring regulatory compliance. Ice cream, one of the most widely consumed frozen desserts worldwide, presents unique safety and quality challenges associated with raw milk handling, pasteurization efficiency, ingredient variability, freezing dynamics, cold-chain management, and post-processing contamination risks. Furthermore, the sustained growth of ice cream production, driven by expanding flavor diversity and increasing consumer demand, has intensified the need for robust and adaptive food safety systems. Therefore, maintaining microbial safety and product stability throughout frozen dairy processing requires highly responsive monitoring and control strategies capable of detecting deviations under continuously changing processing conditions. However, conventional HACCP systems depend heavily on manual inspection, periodic sampling, and operator-based decision-making, which limit their capacity for continuous monitoring, rapid hazard detection, and adaptive process control under dynamic industrial conditions [1].

Recent advances in artificial intelligence (AI), machine learning (ML), and digital monitoring technologies are transforming food safety management by enabling predictive analytics, automated anomaly detection, and intelligent process optimization. AI-assisted systems can process large volumes of production data to identify contamination risks, predict equipment failure, improve traceability, and support data-driven corrective actions. Consequently, these technologies are increasingly being explored as complementary tools that enhance conventional HACCP frameworks in modern food manufacturing systems [2,3,4,5,6].

The internet of things (IoT) has emerged as a critical infrastructure supporting intelligent HACCP implementation through the integration of wireless sensors, cloud-connected monitoring platforms, and automated data-acquisition systems. In dairy processing environments, continuous monitoring of temperature and storage conditions is crucial because process deviations can accelerate microbial growth, compromise product stability, and reduce shelf life. Previous studies have demonstrated that sensor-based systems can improve traceability, process verification, and rapid detection of deviations across food production chains [7,8,9,10,11,12].

In parallel, computer vision and ML technologies are increasingly being investigated for automated inspection, contaminant detection, raw material grading, predictive maintenance, and shelf-life estimation. Advanced deep learning (DL) architectures, including convolutional neural networks (CNNs) and generative adversarial networks (GANs), have further expanded the capabilities of intelligent food safety systems by improving hazard recognition and automated quality assessment in complex processing environments [13]. These approaches offer advantages over conventional monitoring systems, including improved processing speed, scalability, and adaptability to complex industrial datasets.

Although several peer-reviewed contributions have addressed AI applications in food safety and smart manufacturing environments, limited studies have explored the integration of AI-, ML-, and IoT-assisted approaches into HACCP systems specifically designed for ice cream manufacturing. The existing literature largely focuses on general food industry applications, with comparatively limited emphasis on frozen dairy processing environments and their operational challenges. For example, Revelou, Tsakali, Batrinou and Strati [2] evaluated the application of ML to HACCP monitoring but generally focused on foods of animal origin. Naseem and Rizwan [14] addressed AI implementation within the food safety framework but did not explore its application to HACCP or to a specific product category. Similarly, Balta et al. [15] focused on the application of AI technologies rather than the integration into HACCP or ice cream processing lines.

**Figure 1:**
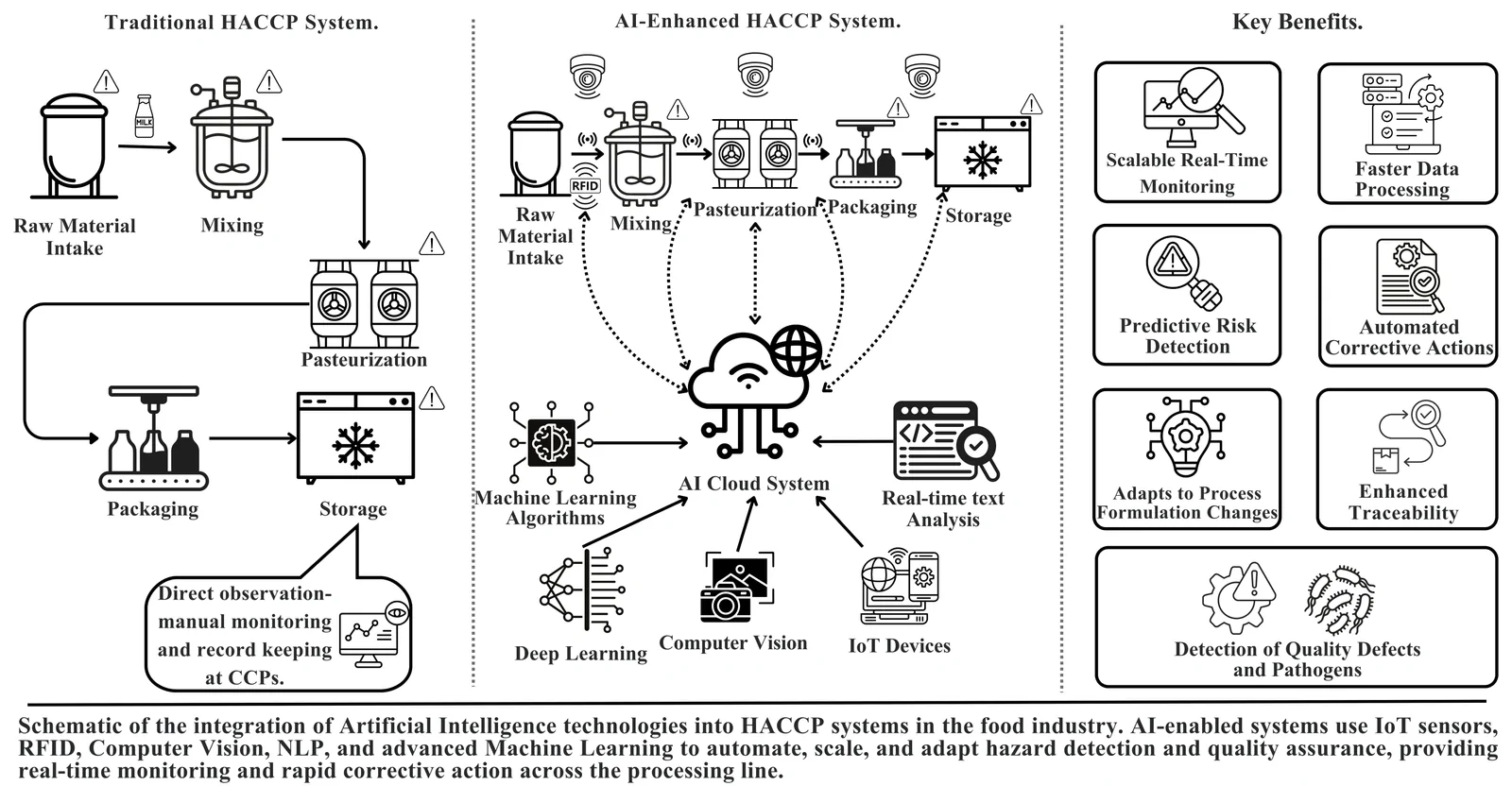
Integration of artificial intelligence (AI) technologies into hazard analysis and critical control point (HACCP) systems for ice cream processing.

Therefore, this review critically evaluates recent advancements in AI-, ML-, and IoT-assisted HACCP systems for ice cream manufacturing, with a focus on intelligent monitoring, predictive hazard detection, automated verification, enhanced traceability, and process optimization at critical control points (CCPs) in frozen dairy production (Figure 1). A systematic literature review was conducted using the Scopus database. The search scope employed combinations of keywords including “HACCP,” “ice cream,” “frozen dairy,” “artificial intelligence,” “machine learning,” “deep learning,” “internet of things,” “natural language processing,” “food safety,” “hazard analysis,” “critical control point,” “traceability,” “computer vision,” “digital twin,” and “predictive maintenance,” resulting in the identification of 142 peer-reviewed sources. Regulatory guidelines from the FAO (Food and Agriculture Organization of the United Nations) and USDA (United States Department of Agriculture) were also utilized to build the framework of the HACCP plan designed in this study. Finally, this review discusses current implementation barriers, technological limitations, and emerging research directions expected to dictate the future of intelligent food safety management in frozen dairy processing systems.

The screening process followed a PRISMA approach. A structured keyword search of the Scopus database returned 4561 records identified, which were screened by title and abstract, and subsequently by full text, against eligibility criteria: relevance to HACCP, AI/ML/IoT applications and dairy or frozen dessert processing. Records were excluded where the primary focus was unrelated to food safety or quality monitoring, where the full text was unavailable, or where the content was duplicated across search terms. This search was supplemented by additional relevant records identified through Google Scholar and manual screening of the reference lists of key articles, yielding the 142 peer-reviewed sources retained in this review.

## 2. Core Technologies for an Intelligent HACCP System

### 2.1. Data Acquisition: IoT

The objective of IoT technologies is to collect and transfer data regarding process conditions or product characteristics through sensors to provide real-time information on the possible variations that may occur across the processing line [8]. For example, a previous study used IoT technologies to ensure food safety and quality assurance by employing Arduino control to collect information regarding food storage conditions such as temperature, humidity, and methane level [16]. The data collected was transmitted via Bluetooth to an application that enabled facile data visualization and anomaly detection [16]. Although these environmental parameters are important for monitoring storage conditions, dairy quality assessment also requires the evaluation of product-related variables, including pH, titratable acidity, microbial load, and other physicochemical indicators. Depending on the processing stage and the available sensing technology, these parameters may be monitored using in-line sensors and integrated into AI-based models for real-time quality assessment and anomaly detection. At the raw milk receiving stage, AI-assisted monitoring can also support the early identification of both microbiological and chemical hazards before milk enters storage silos. Data generated from rapid microbiological screening methods, biosensors, spectroscopic techniques (e.g., FT-IR or Raman spectroscopy), electronic nose systems, and routine quality analyses can be integrated into machine learning models to identify abnormal samples containing pathogenic microorganisms, elevated somatic cell counts associated with mastitis, antibiotic or veterinary drug residues, aflatoxin M1, pesticide residues, heavy metals, or other contaminants. Milk batches predicted to pose unacceptable safety or quality risks can then be flagged for confirmatory laboratory testing or rejected before entering the processing line, thereby strengthening preventive HACCP control at the raw material receiving stage. Considering this, an effective IoT technology generally comprises three important aspects: sensors, communication technologies, and cloud computing platforms [17]. After acquisition, data are typically transferred through Wi-Fi or other transmission mechanisms to cloud-based systems for storage. These records can support traceability in case of a pathogen outbreak, processing condition deviation, or product quality defect [17].

**Figure 2:**
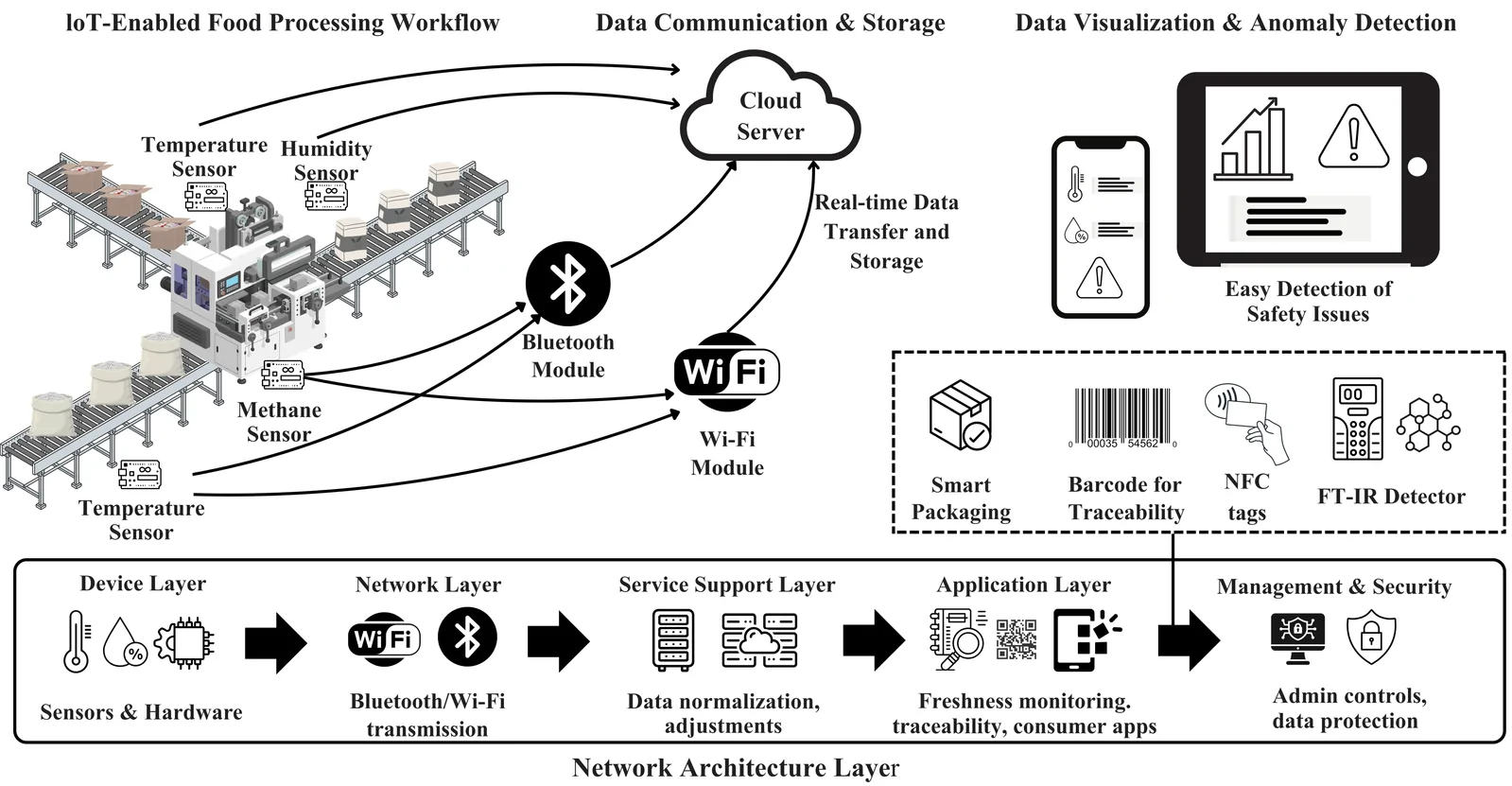
Architecture of an internet of things (IoT)-based monitoring system with sensor layers and cloud integration. Icons on the mobile display represent, from top to bottom: temperature/sensor readings, real-time data alerts, and a safety-deviation warning, illustrating the type of information relayed to end users for rapid detection of safety issues.

**Figure 3:**
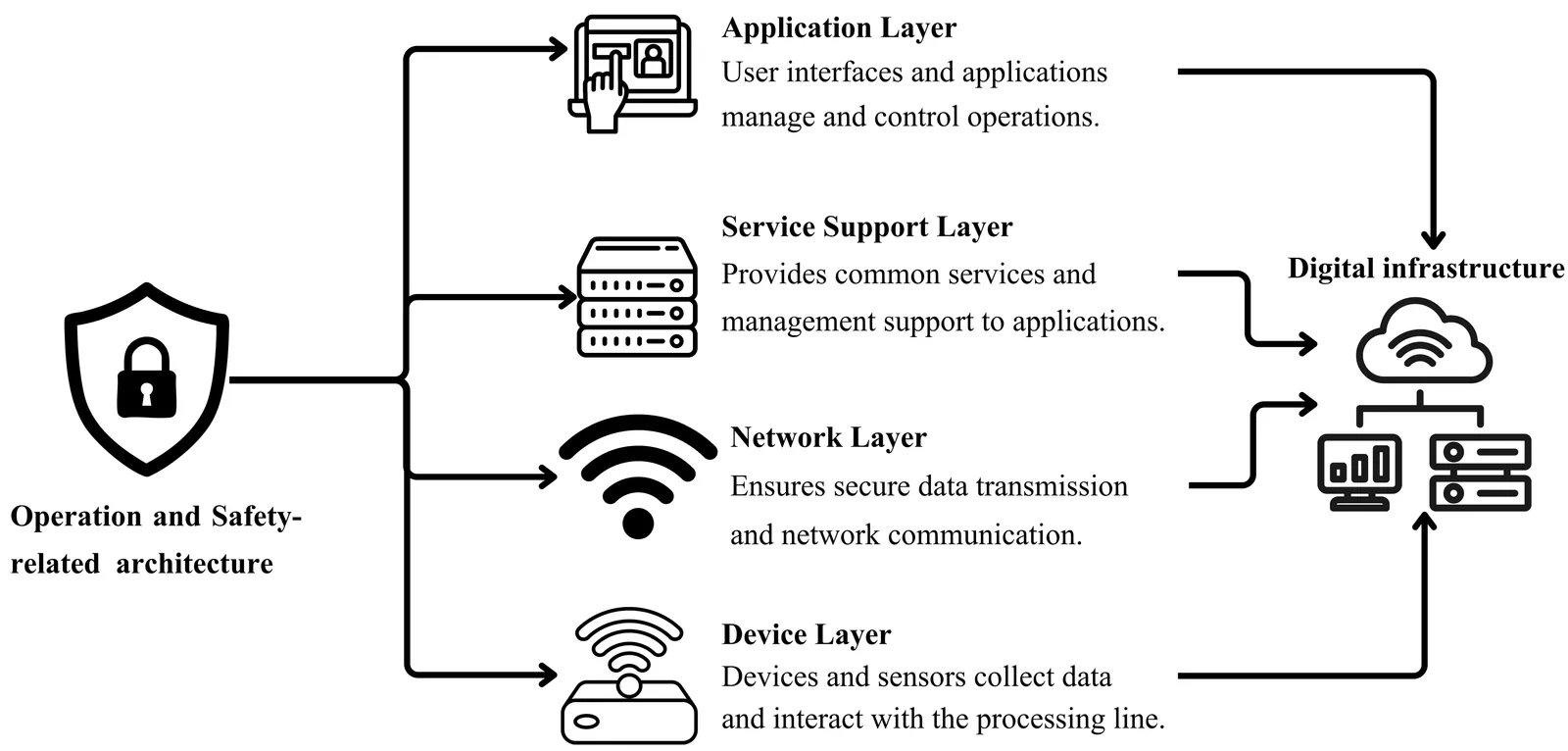
IoT infrastructure for food safety monitoring, comprising the application, service support, network, and device layers that support operational and safety-related processes. Adapted with permission from Ref. [8].

As illustrated in Figure 2 and Figure 3, the architectures of IoT networks are alternatively classified into a multilayer framework consisting of a device layer, network layer, service support layer, application layer, and management and security layers [8]. The device and network layers are similar to the previously described physical hardware required for data collection and transfer to the cloud. The service support layer is relevant for data normalization and adjustments of processing conditions [8]. The application layer encompasses the potential applications of IoT technologies, including the limitations, opportunities, and challenges. The management and security layers control the administrative aspects of integrating these technologies into a processing line [8]. In a prior study, IoT technologies were integrated with smart packaging and sensors to evaluate product freshness and barcodes in order to improve traceability within the processing line [18]. The authors proposed the implementation of near-field communication (NFC) tags in packaging to enable consumers to verify product freshness directly from their mobile phones, as well as the use of detection technologies such as Fourier-transform infrared (FT-IR) to identify the source of animal-derived foods [18].

### 2.2. Automated Inspection: Computer Vision

**Figure 4:**
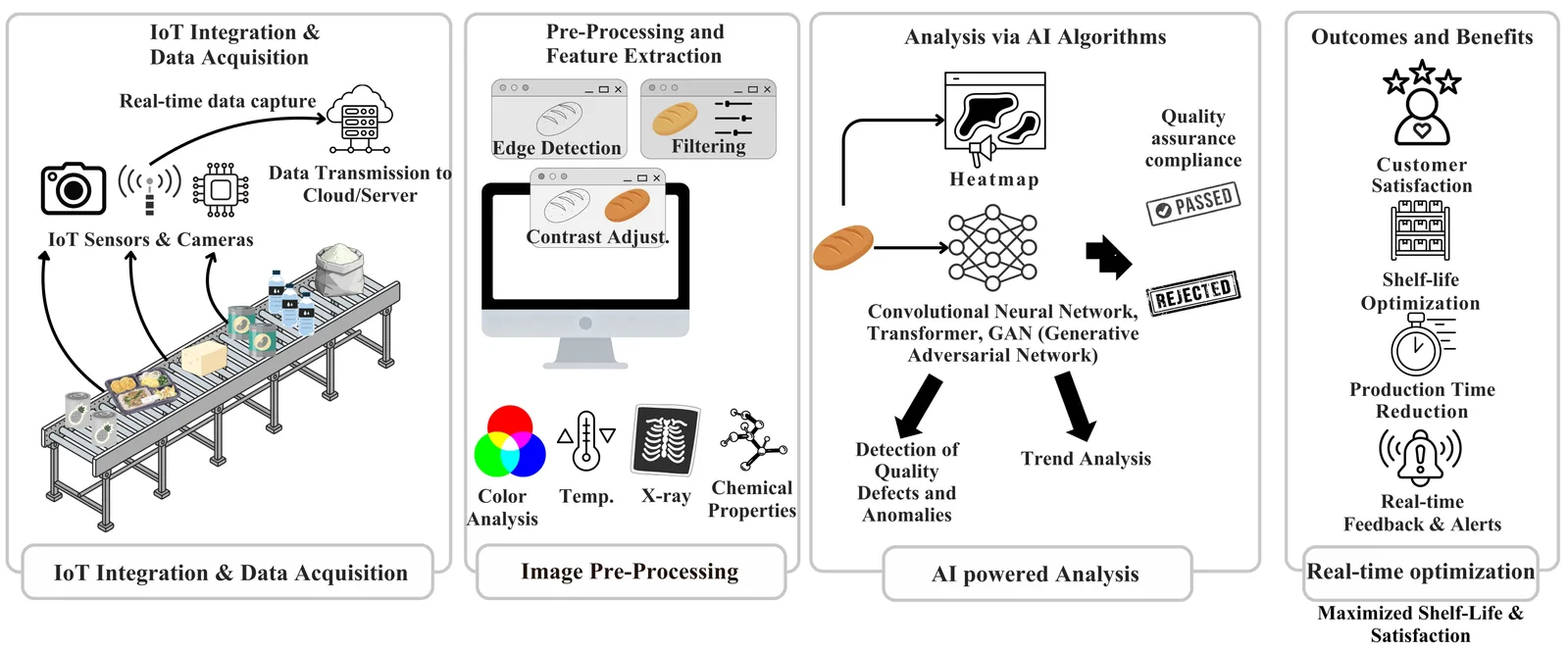
Schematic workflow of an integrated computer vision and AI system for food quality assessment, highlighting stages from IoT data acquisition to real-time optimization.

Computer vision represents an application of sensing technologies. It relies on sensors and image-capturing devices for data collection, data analysis, and the prediction of emerging hazards or quality defects [19]. However, studies highlight that computer vision is most effective when integrated with AI technologies such as CNNs, transformer-based models, and GANs [20]. Computer vision workflows consist of four main stages: acquisition, preprocessing, analysis, and interpretation [21]. The equipment required for applying computer vision to most processing lines typically comprises a camera and image-editing software that enables the acquired data to be processed in order to remove lighting or background interference [21] (Figure 4). Most computer vision technologies are used to collect red–green–blue (RGB) color information. However, recent studies have expanded these technologies beyond conventional color analysis to include X-ray imaging for internal defect detection, particle-size determination for structural characterization, measurements of foam-forming capacity to evaluate aeration properties, and interpretation of enzyme-linked immunosorbent assay (ELISA)-based colorimetric signals for rapid biochemical analysis. These examples illustrate the broad analytical capabilities of computer vision and AI-assisted sensing systems. However, their application to ice cream manufacturing requires validation against dairy-specific quality attributes such as fat globule size, ice crystal size, overrun, emulsion stability, and thermal quality changes relevant to HACCP monitoring [20,21]. For example, colorimetric RGB and hyperspectral imaging have been applied to detect Maillard-related discoloration, while electronic nose and volatile compound sensing approaches have been used to detect cooker or burnt off-flavors resulting from thermal abuse during pasteurization; these outputs can serve as additional input features for AI-based quality classification models [20,21]. It should be noted that these particular examples were reported in studies on non-dairy beverages and are cited here to illustrate the broader range of physicochemical attributes that computer vision has been extended beyond color. Their direct application to ice cream manufacturing would require validation against dairy-relevant targets such as fat globule size, ice crystal size, and overrun/foam structure.

RGB camera imaging and multi-image acquisition generate large image datasets by continuously capturing product surface characteristics under standardized lighting conditions from multiple viewpoints or at successive processing stages. These image datasets are subsequently processed to extract color, texture, and structural features that serve as inputs for AI-based quality prediction models. In ice cream manufacturing, these imaging systems are particularly valuable during pasteurization for monitoring heat-induced color changes, during homogenization for evaluating emulsion uniformity, during freezing for assessing ice crystal development, and during final product inspection for detecting visual defects and ensuring product consistency.

Multi-angle or multi-image RGB acquisition improves robustness to lighting and orientation variations for surface defect and color assessment. Among the processing stages discussed in this review, raw material reception and packaging are considered to benefit the most from RGB-based inspection, given the practical importance of decision-making at these points. However, before any such model is deployed for CCP monitoring, standard model validation practice would apply: partitioning data into training, validation and test sets (cross-validation), together with external validation against confirmatory laboratory or reference data.

Considering these applications, computer vision must be adjusted to the parameters of the product being assessed. Upon acquisition, the data are processed to remove interference caused by shadows, variations, or overall image noise. For example, methods such as edge detection, filtering, and contrast adjustment can be used to prepare the images for further analysis [20]. Additionally, the properties to be measured, such as texture, shape, or color, should be determined based on the design of the processing line [20]. To improve the detection limits of sensors and cameras, AI algorithms, such as CNNs, can be implemented to analyze the current properties of the product, offer possible improvements, identify quality defects, and support prevention measures in the processing line [22]. In a study on the detection and image recognition of Central Asian foods, the researchers concluded that computer vision technologies are powerful tools for characterizing the type of product being processed, while AI models can aid in directly processing the image source and prioritizing the most important features for controlling the sensory properties of the product [22]. Overall, these approaches enable the real-time detection of anomalies and quality defects and the analysis of processing parameter trends to improve consumer satisfaction, shelf-life optimization, and production time management [22].

### 2.3. Predictive Analytics: ML, DL, and Natural Language Processing

**Figure 5:**
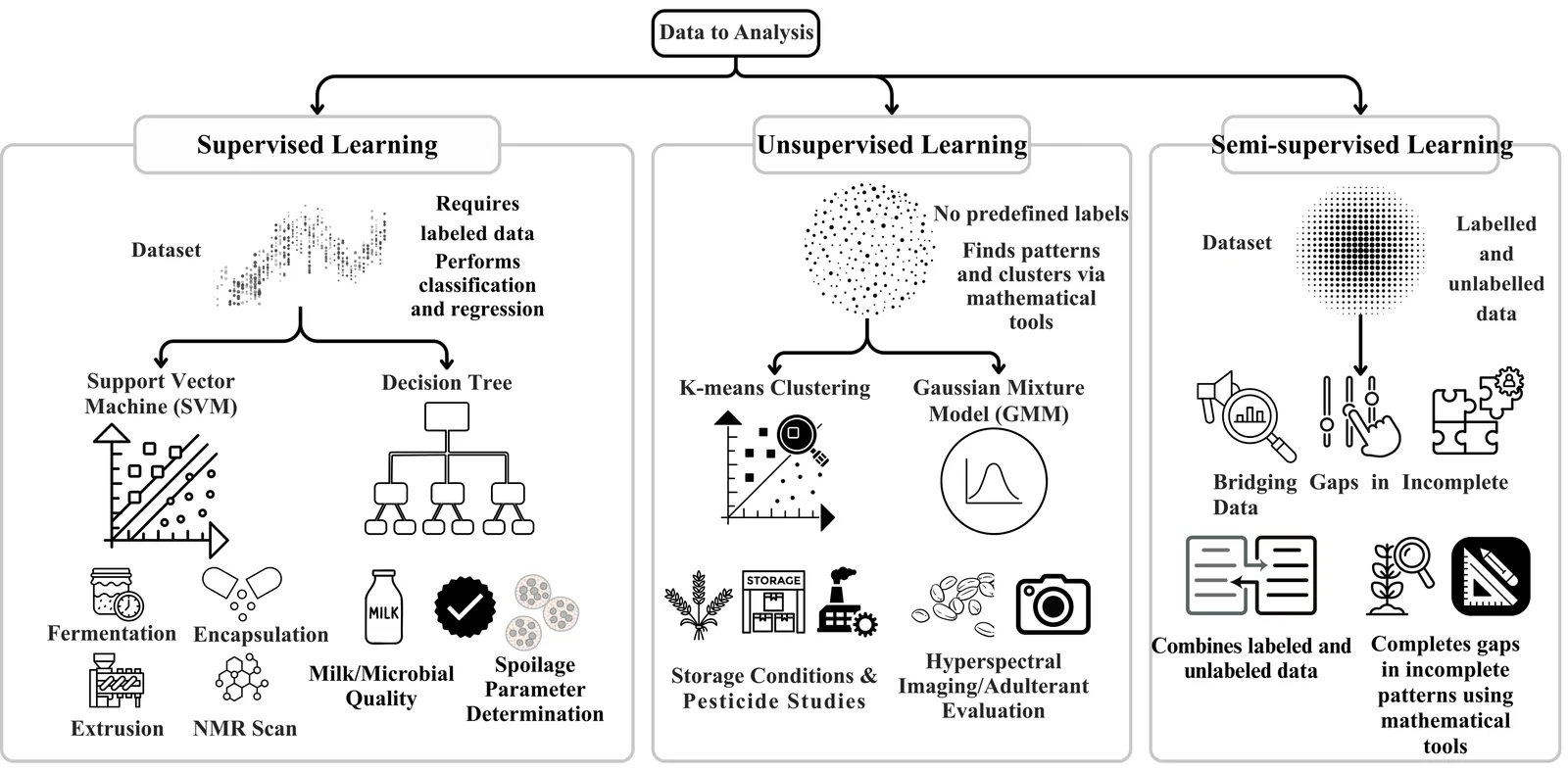
Overview of machine learning (ML) algorithm types (supervised, unsupervised, and semi-supervised) and their applications in food science, including fermentation monitoring and adulterant detection.

ML algorithms bridge the gap between empirical data acquisition and predictive modeling in food processing, enabling shelf-life estimation, contamination detection, and processing optimization [23]. Studies generally categorize ML algorithms into three types: supervised, unsupervised, and semi-supervised (Figure 5). The selection of these paradigms depends on the structure of the available data, the linearity or nonlinearity of the dataset, and the specific problem to be solved [24]. Supervised models use datasets with defined patterns or labels to learn relationships and enable predictions with low error rates. In contrast, unsupervised models operate with unlabeled or undefined datasets and utilize mathematical tools and associations to provide reliable data on measured parameters. Semi-supervised models combine both approaches to address incomplete datasets [24].

In this context, contamination detection encompasses two distinct hazard categories that require different analytical approaches: chemical hazards (e.g., pesticide residues, adulterants, veterinary drug residues, and heavy metals), which are typically screened using spectroscopic methods such as FT-IR/NMR coupled with chemical models, and microbial hazards (e.g., pathogenic bacteria), which are typically screened using rapid biosensor or molecular methods. AI-assisted classification of these screening outputs at raw material reception supports an accept, hold, or reject decision for incoming raw milk before it proceeds to storage.

Relevant ML models include support vector machines (SVMs) and decision trees for supervised learning and K-means clustering and Gaussian mixture models for unsupervised learning [23]. SVMs can be used for both regression and classification, making them suitable for datasets related to unit operations such as fermentation, encapsulation, extrusion, and adulterant detection using techniques such as nuclear magnetic resonance (NMR) [25]. Similarly, decision trees require defined labels to create a fitting model. Specifically, they utilize hierarchical splits and employ the averages to reach a reliable decision [2,23]. For example, this model has been previously used to classify microbial parameters of milk, determine the quality grade, and detect bacterial spoilage during processing [2].

**Figure 6:**
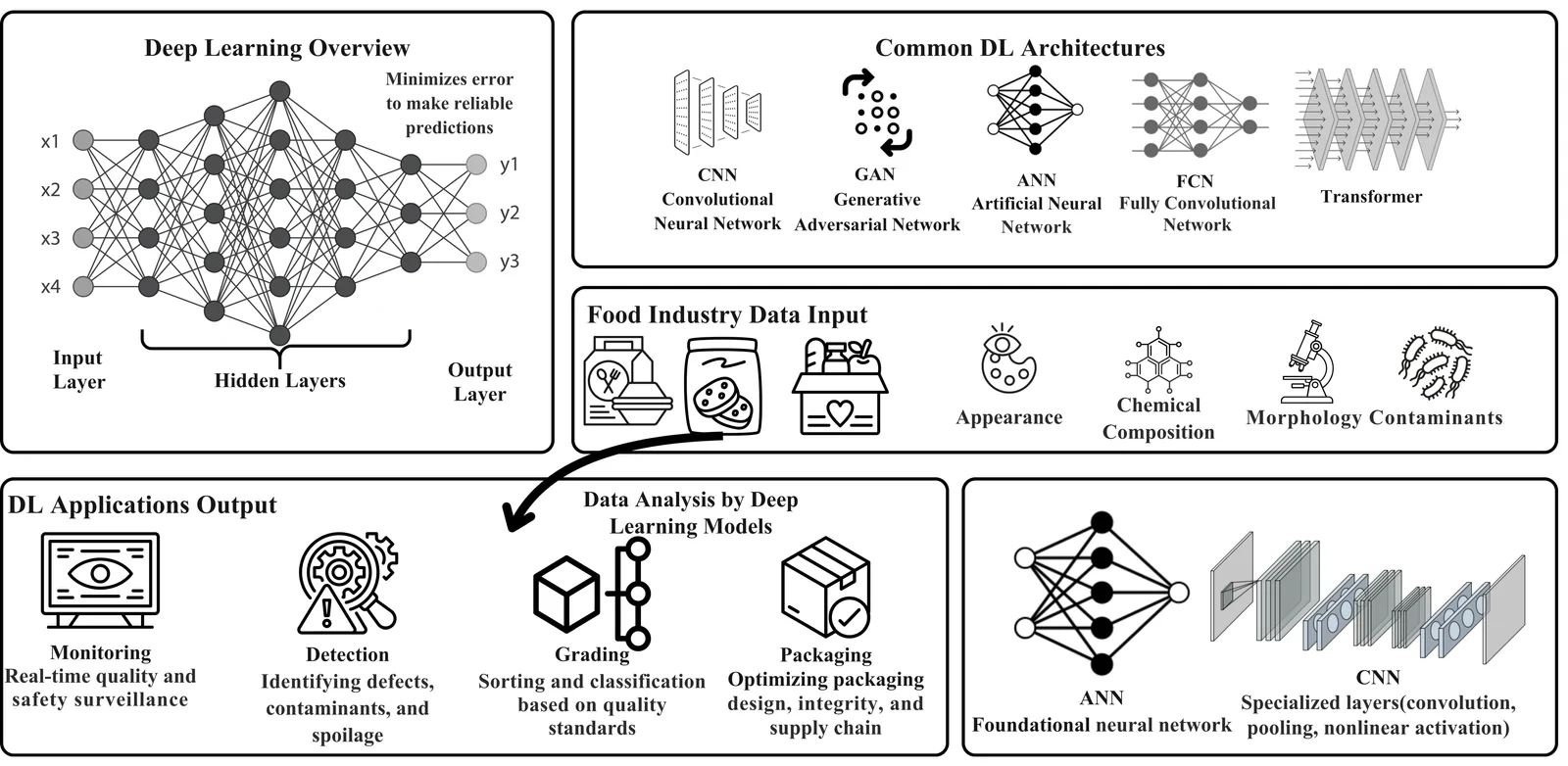
Applications of deep learning (DL) architectures in the food industry.

Unsupervised models such as K-means clustering and Gaussian mixture models identify patterns in unlabeled data to analyze storage conditions and detect adulterants through hyperspectral imaging [26,27]. Concurrently, models such as CNNs, GANs, artificial neural networks, fully convolutional networks, and transformers are being rapidly implemented in the food industry (Figure 6). Specifically, these models analyze data on raw materials such as fruits, fish, grains, and vegetables, focusing on variables related to appearance, chemical composition, morphology, or the presence of contaminants [20,28]. It should be noted that most published hyperspectral imaging and ANN applications of this kind have been developed for more stable or semi-perishable commodities. Direct transfer to raw milk requires further validation given milk’s higher compositional variability and faster spoilage kinetics, and this is noted as a research gap in Section 5.

Natural language processing (NLP) comprises technologies designed to interpret human language from sources such as reports, historical data, regulations, and texts to collect information on determined parameters [29]. The extracted information is subsequently applied to decision-making within the processing line. Although the implementation of NLP in the food industry is still developing, the main applications include retrieving information on labeling policies, regulations governing additives, critical contaminant limits, antecedents of pathogen outbreaks, and food fraud reports [29]. Moreover, NLP algorithms can be used to enhance the reliability of DL models to establish theoretical frameworks and predictions through language interpretation [30]. One notable application involves using NLP algorithms to analyze the nutrient concentration in certain food samples and categorize the extent of their processing [30].

For spoilage-related classification tasks of this kind, differentiating pathogenic microorganisms from milk macrocomponents (e.g., proteins and fats) relies on training models against spectral or imaging signatures specific to microbial cell structures, rather than general turbidity or compositional signals.

## 3. AI and ML Applications Across HACCP Fundamental Principles

Considering the fundamental principles of HACCP, recent studies have focused on applying IoT, computer vision, ML, DL, and NLP while developing HACCP plans, as summarized in Table 1 [31,32]. Specifically, ML algorithms can read and interpret data from various reports (e.g., safety logs, consumer reports, historic data, and anomaly reports) to identify trends or correlations among possible sources of contamination and how they can be prevented in the future [14]. However, these algorithms should be implemented alongside predictive algorithms such as decision trees or SVMs, which label critical safety parameters and determine the most prevalent risks throughout the processing line [14]. For effective implementation of AI-assisted algorithms, the microbial and chemical composition of the raw material is crucial for determining whether a stage should be classified as a CCP or critical point (CP). In this regard, correlations among microbial indicators (such as mold, yeast, and lactic acid bacteria), pathogens (e.g., *Staphylococcus aureus*, *Listeria monocytogenes*, *Salmonella*), and chemical contaminants (e.g., lead, arsenic, nitrite and mercury) provide valuable information for employing AI technologies to identify the process stages that are critical to product safety [33]. Building on this concept, studies have proposed AI-assisted approaches involving classification of data and simulation of pasteurization parameters, such as flow rate, pH, temperature, pressure, and conductivity, to optimize risk detection and improve overall product safety [34,35]. Collectively, AI technologies offer significant advantages by enhancing data acquisition, improving data reliability, and providing deeper insights to verify whether food safety hazards are effectively controlled [36,37].

**Table 1:** HACCP fundamental principles and corresponding AI implementation technologies.

HACCP Principle	Principle Objective	AI Implementation
Hazard analysis	Identify and evaluate pathogens (such as *Salmonella*, *Listeria monocytogenes,* and *E. coli*), chemical residues, metals, or any foreign agent across each unit operation during processing [31,38].	Electrochemical, colorimetric, and fluorescent IoT sensors [39]. Coupled ML and DL architectures for analysis of reports (safety logs, consumer reports, historic data, anomaly reports, etc.) [31]. Visual data acquisition systems and hyperspectral imaging for adulterant identification [40,41]. Decision trees or support vector machines (SVMs) for risk assessment [14]. Convolutional neural networks (CNNs) for identifying optimal parameter deviations [42].
Determination of critical control points (CCPs)	Determine which unit operations constitute CP and CCPs to ensure that proper safety practices are applied accordingly [32,43].	Random forests or SVMs for hazard detection and analysis [44]. Text data mining algorithms for extracting international and local safety guidelines [45]. Hard-O-Tronic AI (HOT-AI) for critical stage determination [41]. Naive Bayes models for predicting the emergence of hazards for risk management [46]. Models for correlating microbial parameters for CCP determination
Determination of critical limits	Establish the minimum and maximum allowable values for physical, chemical, and biological parameters at the determined CCPs to prevent, eliminate, or reduce food safety hazards [32].	NLP models for automated extraction of critical parameters [29]. SVMs and multiple linear regression models for critical limit determination and hazard occurrence quantification. [47] Digital twins for simulation and modeling of the processing line [19,34].
Monitoring	Ensure that values remain within the critical limit through the documentation of acquisition frequency, collection methods, and storage locations [40].	IoT sensors and ML algorithms for automated data collection [40]. Amazon Web Services (AWS) for optimal data storage; Wi-Fi and long-range wide-area network (LoRaWAN) for communication [7,36]. IoT sensors for measuring temperature, light, air quality, ammonia concentration, and pH [40]. Explainable AI (XAI) models for correlating organic compound parameters [37]. ML models for adulteration identification [48]. Image acquisition for modeling shape retention and melting rates [49]
Corrective actions	Deploy documentation, cause analysis, and resolution protocols upon detection of critical deviations [38,44].	IoT sensors for data collection and interpretation [43]. AI analysis for cause and response protocol analysis [43]. DL algorithms for analyzing deviation response protocols Spectral processing techniques for detecting and implementing corrective protocols [50].
Verification and validation	Evaluate the HACCP plan to ensure its efficacy in minimizing the recurrence of an identified food safety hazard [46].	Shapley additive explanations (SHAP) for prediction explanation and applicability [51]. Monte Carlo dropout for model reliability assessment [51]. Blockchain and Gaia-X compliant architectures for data security [52,53]. MIDAS (Modular ice cream factory dataset on anomalies in sensors) architecture for anomaly injection and validation of corrective actions [54]. ML models for ISO2200:2018compliance [55,56]
Documentation	Store HACCP-related documents for accessibility, future decision-making, audits, and traceability purposes [38].	AWS cloud for sensor data logging [57]. Blockchain framework for data safety in compliance with ISO 22005:2007 [57,58]. Gaia-X and DataOps as a storage framework [59]. Radio frequency identification (RFID) and Near-field communication (NFC) tagging for lot identification [38].

## 4. Case Study: Design of an AI-Enhanced HACCP System for Ice Cream Manufacturing

The AI-enhanced HACCP framework presented in this section is a conceptual design synthesized from the peer-reviewed literature and regulatory guidelines reviewed above. It has not been implemented or validated in an industrial production environment, and future studies using real industrial production data are required to validate its practical effectiveness.

**Figure 7:**
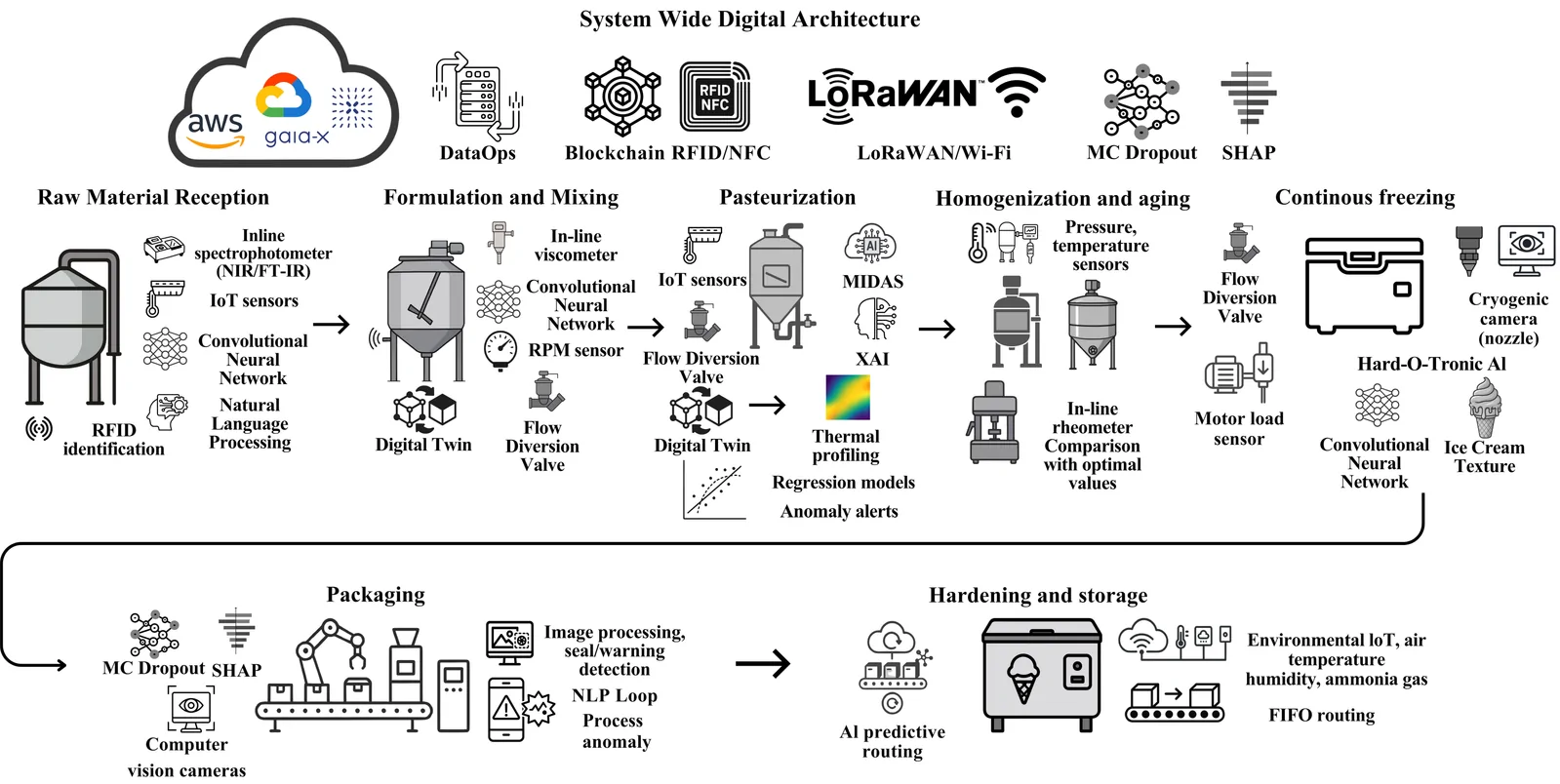
Process flow diagram of an intelligent smart ice cream manufacturing system illustrating the digital architecture overlay and unit operations.

To systematically integrate AI-assisted technologies across the different HACCP fundamental principles for ice cream manufacturing, the overall architectural framework was first defined by specifying the AI-assisted technologies assigned to each unit operation across the processing line (Figure 7). Subsequently, hazard analysis was conducted as depicted in Table 2, and the CCPs, critical limits, and monitoring procedures were then determined, as detailed in Table 3 and Table 4. The verification, validation, and documentation protocols are presented in Table 5.

### 4.1. Process Flow Mapping

#### 4.1.1. System-Wide Digital Architecture

As established in the theoretical framework, the transition toward an intelligent ice cream processing line requires robust, macro-level digital architectures that ensure transparency, traceability, and overall product safety. For example, cloud-based infrastructures such as Amazon Web Services (AWS) and Google Cloud serve as foundational tools for real-time data collection and secure storage [52]. Gaia-X complements the data collection framework by aligning it with local food safety guidelines, ensuring data integrity through the prevention of unauthorized manipulation, and creating a centralized hub where data can be easily retrieved and analyzed [52,60].

Blockchain, in conjunction with Gaia-X, provides enhanced data security, traceability insights, and rapid recall decision-making, bridging storage architectures with NFC and radio frequency identification (RFID) tags to guarantee lot-level integrity across unit operations [53]. Specifically, blockchain bridges the gap between data storage architectures and traceability-focused sensors such as NFC and RFID tags [61]. These technologies provide precise lot identification at each unit operation, guaranteeing product integrity, enabling appropriate corrective action, and facilitating the evaluation of the overall effectiveness of the HACCP plan [61].

Finally, long-range wide-area network (LoRaWAN) and Wi-Fi modules serve as the critical communication backbone for implementing an intelligent processing line [62]. IoT sensors and devices generally require these wireless communication technologies to transmit data securely, allowing the system to automatically detect deviations from critical limits and determine how the subsequent food safety hazard can be reduced, controlled, or eliminated [62]. Both protocols operate simultaneously, ensuring continuous sensor communication even during network disruptions [62].

The ML models integrated across the processing line require periodic fine-tuning to ensure that their predictions accurately reflect real-time processing conditions. Without regular calibration, these models may generate false positives or trigger corrective actions under normal operating conditions. To address this, Monte Carlo dropout has been proposed as a standardized uncertainty quantification method that can be applied during each unit operation [63]. By randomly deactivating neurons during inference, Monte Carlo dropout produces a distribution of predictions rather than a single output, enabling the system to distinguish reliable predictions from uncertain ones before making a decision [63]. Complementary tools such as Shapley additive explanations (SHAP) can further validate model outputs (i.e., the obtained values). When combined with explainable AI (XAI), SHAP has been reported to achieve 95% accuracy in the classification of data obtained through hyperspectral imaging of anthocyanin content in sweet potatoes [64]. Nonetheless, the model could be applied to ice cream manufacturing since it demonstrates the potential to perform quality assurance analysis through detailed pixel visualization of the raw ingredients to the final product.

#### 4.1.2. Raw Material Reception

During raw material reception, maintaining ingredient integrity is the primary objective governing all associated operations. To achieve this, in-line spectroscopic sensors (such as NMR or FT-IR analyzers) should be installed either on the transport tankers or within the storage silos containing the raw materials (e.g., milk, sugar, cream, stabilizers, and flavor-related additives) [65]. IoT temperature sensors must be deployed to continuously monitor highly perishable, pathogen-sensitive materials (such as milk and cream). The data obtained from these sensors is then processed by a CNN trained to verify if the received material complies with regulations regarding adulterants [66]. NLP models should be integrated to cross-reference incoming ingredient specifications with current local food safety regulations [67]. Finally, for comprehensive traceability, each received material must be assigned an RFID tag containing the lot number, reception date, expiry date, supplier information, and any manual observations logged by the supply chain team upon arrival [68].

#### 4.1.3. Mixing

In the mixing stage, all previously inspected raw materials are incorporated, requiring precise control of physicochemical parameters to ensure the safety and consistency of the final product. To optimize this process, an in-line viscometer should be implemented to continuously monitor viscosity, as deviations from critical limits may indicate incorrect ingredient ratios, incomplete incorporation of stabilizers, or potential contamination [69]. These data serve as critical inputs for downstream CNN models trained to predict ice crystal distribution during freezing, establishing a data-driven sequence between the mixing conditions and the final product [70]. If a deviation is detected, a flow diversion valve (FDV) actively diverts and isolates the affected batch before it advances to the next unit operation. A level control sensor must be installed within the mixing vessel to ensure batch size consistency and significantly reduce the occurrence of overflow events [36]. Similarly, a revolutions per minute (RPM) sensor should be integrated with the machine to ensure that the mixing speed remains within the established processing conditions and to detect discrepancies between equipment setpoints and sensor readings [71].

#### 4.1.4. Pasteurization

The primary objective of the pasteurization stage is to ensure that the thermal treatment effectively eliminates pathogens (e.g., *Listeria monocytogenes*, *Salmonella*, and *E. coli*). Therefore, redundant temperature and flow sensors must be implemented to monitor and control these parameters to ensure proper maintenance of the critical limits throughout the process [72]. Similar to the case of the previous stage, an FDV should be coupled to the pasteurizer to ensure that upon temperature deviation detection, the affected batch can be isolated or redirected while the corrective action is executed by the previously trained ML algorithms [73]. Specifically, XAI serves as a powerful tool to translate these algorithmic decisions into transparent data logs and prove to auditors that the corrective actions strictly comply with local food safety regulations [74]. Another relevant tool for verifying overall process efficiency is a digital twin coupled with Ansys Fluent, which connects the different process unit operations and enables temperature profiling to detect deviations of sensor readings from the actual equipment operating conditions [75]. It should be clarified that Ansys Fluent is applied here as a computational fluid dynamics (CFD) tool to simulate flow and identify low-shear, low-flow regions of equipment and pipework that are prone to biofilm formation, rather than as a direct biofilm-detection sensor. Confirmation of actual biofilm presence still requires microbiological sampling and analysis. In addition to microbiological safety, excessive thermal exposure can produce cooked or burnt flavor notes and slight yellow discoloration through heat-induced reactions. Computer-vision color measurements (RGB or hyperspectral imaging) and electronic-nose or volatile compound sensing can therefore be incorporated as supplementary input for features for AI models that classify thermal quality and detect potential overprocessing [20,21].

#### 4.1.5. Homogenization and Aging

The homogenization and aging stage primarily aims to ensure that the mix develops a stable, uniform emulsion and that all phases are properly incorporated, preventing destabilization during subsequent processing stages. To achieve this, IoT sensors are deployed to continuously monitor critical parameters, particularly the pressure across the homogenizer pass, as well as the pH and temperature within the aging vats [48]. Another important aspect of the aging stage is the assessment of the structural state of the mix. For this purpose, an in-line rheometer can be installed to detect if there are unusual changes in texture and verify if the mix complies with previous optimal values [76]. ML regression models and time-series analysis are implemented to compare real-time acquired data against historical data of product batches that exhibit optimal physicochemical characteristics [77]. This enables the prediction and adjustment of fat-crystallization and hydration time while mitigating associated food safety hazards.

#### 4.1.6. Continuous Freezing

Continuous freezing is a stage with challenges specific to ice cream, distinguishing it from general food processing: ice crystal nucleation and growth during freezing and subsequent temperature fluctuations directly affect product texture, and must be minimized to preserve smoothness; overrun (the incorporation of air into the mix) must be controlled within a target range to achieve the desired body and yield without compromising structural stability; and frozen storage stability depends on maintaining consistent sub-freezing temperatures throughout the cold chain, since even brief fluctuations can promote ice recrystallization and textural degradation. The AI and IoT-based monitoring approaches discussed below are presented with these ice cream-specific quality attributes in mind, rather than as generic process controls applicable to any frozen or general food product.

Continuous freezing is required to rapidly freeze the mix, prevent excessive ice crystal growth beyond the specified limits, and ensure a smooth consistency and an optimal overrun value. Considering that extrusion temperature and flow are critical processing parameters, redundant thermal probes must be implemented within the freezing barrel [78]. A cryogenic computer vision camera should be installed at the extrusion nozzle to capture the surface texture of the product [79]. These high-speed captured images are processed by a CNN trained to evaluate the suitability of the ice crystal size distribution. To prevent network latency, the CNN should operate via edge computing to correlate the obtained visual data with the final overrun values. This edge architecture interfaces directly with AI-driven control models such as Hard-O-Tronic AI (HOT-AI) frameworks, which dynamically modulate refrigerant flow and air injection to optimize textural properties [80]. To ensure proper mechanical operation of the freezer, a dasher motor load sensor should be coupled to the equipment. ML models analyze this internal viscosity data to detect deviations, verify that corrective actions are executed appropriately, and establish accurate predictive maintenance protocols. A mass flow meter installed at the extruder nozzle continuously monitors the dispensed volume, ensuring batch consistency. If there are operating parameter deviations, the FDV is automatically activated by the AI architecture to isolate the noncompliant product. Within the HACCP framework proposed in this review, HOT-AI refers to an AI-driven digital twin control layer that continuously compares real-time process sensor data against the critical limits established for the corresponding CCPs (Section 4.2.3) and feeds deviation signals into the monitoring and corrective action logic described in Section 4.2.2, Section 4.2.3 and Section 4.2.4, rather than operating as a stand-alone strategy.

#### 4.1.7. Packaging

The primary objective of the packaging stage is to ensure that the ice cream is precisely dispensed into tubs and that the hermetic seals are installed correctly, thereby preventing subsequent contamination or unauthorized product adulteration. High-speed computer vision cameras installed across the primary and secondary packaging lines verify label, tub, and seal positioning [81]. For this purpose, AI models must be trained to accurately segment and identify overlapping or touching packaging objects, allowing the system to instantly detect any noncompliance during this stage and trigger the immediate activation of the corrective action protocols [20]. The integration of NLP models is also crucial to analyze noncompliance reports from consumers, distributors, or retailers. This creates a closed-loop feedback system that effectively detects the origin of an anomaly and generates predictive alerts to prevent future disruptions to the packaging protocol [82]. Finally, an automated metal detector must be installed at the end of the sealing module to identify physical contaminants within the final product and trigger the rejection and isolation of affected batches [83].

#### 4.1.8. Hardening and Storage

The hardening and storage stage represents the final unit operations in the ice cream processing line. This stage focuses on maintaining optimal environmental conditions (e.g., temperature, humidity, and product spatial distribution within the storage chambers) to prevent product contamination, early spoilage, or the occurrence of any associated food safety hazard. Similar to the case of prior stages, IoT sensors that measure air temperature, humidity, and ammonia gas levels must be installed throughout the storage chambers to continuously verify optimal conditions, detect signs of early spoilage, or detect potential refrigerant leaks [84]. A digital twin powered by Ansys Fluent enables real-time three-dimensional temperature profiling, allowing the identification of thermal dead zones and verification of product rotation protocols [85]. Specifically, ML algorithms can generate dynamic inventory routing maps to predict the optimal placement and rotation of batches in order to strictly enforce first-in-first-out (FIFO) protocols [86].

### 4.2. Building the Intelligent HACCP Plan

#### 4.2.1. Hazard Analysis

Throughout ice cream processing, associated biological, chemical, and physical hazards must be controlled within acceptable levels to prevent pathogen proliferation and foreign object contamination. As depicted in Table 2, the product is exposed to multiple contamination sources, starting from raw material reception to the hardening and storage stage. The primary chemical hazards include residual detergents, cleaning solutions, raw material adulterants, and allergen cross-contamination from prior batches. The likelihood of recurrence increases under continuous processing and demanding production schedules [10,87]. Equipment aging represents another source of chemical hazards, as structural damage can lead to cross-contamination between batches through refrigerant leaks, mechanical lubricant infiltration, or food-grade oil leaks [88]. Packaging components also represent an important chemical hazard source because damaged materials may allow food-grade inks and adhesives to migrate into the product [89,90]. To address these hazards, AI models, network architectures, and IoT hardware are integrated throughout the processing line. Specifically, FT-IR and NMR spectrometers, sensor data comparison protocols, cloud storage, DL models for deviation detection, NLP for consumer and retailer feedback analysis, IoT sensors for equipment state monitoring or ammonia gas detection, viscometers, and computer vision cameras collectively support chemical hazard control to prevent product contamination and future recalls [5,88,91].

As summarized in Table 2, FT-IR/NMR are applied primarily for rapid physicochemical characterization of incoming milk and cream (composition, adulteration, and freshness indicators), while detection of specific foodborne pathogens (e.g., *Escherichia coli*, *Salmonella*, *Listeria monocytogenes*) relies on complementary targeted biosensor or molecular methods with organism-specific signatures, rather than on total viable count alone. This distinguishes pathogen screening from generic microbial load monitoring and from beneficial or starter microorganisms present in cultured or probiotic formulations, which are not flagged by these targeted assays.

Physical hazards across the processing line are associated with foreign objects introduced in the product stream. These include metal shards, screws, hard plastic fragments, equipment components, gasket remains, glass, environmental debris, manual handling contaminants (e.g., hair and nails), and damaged packaging pieces, all of which pose significant product safety risks [92]. To control these hazards, a combination of ML models, NLP, hyperspectral imaging, IoT sensors that monitor mechanical stress through acoustic and RPM measurements, SHAP-based models for fine-tuning, RFID tags, and X-ray sensors can be implemented to reduce the probability of hazard recurrence and minimize compromised batches and noncompliance reports from consumers and retailers [93,94,95,96].

Biological hazards are also critical throughout the processing line. Pathogenic bacteria such as *Listeria monocytogenes*, *Salmonella* spp., *Staphylococcus aureus*, and *E. coli* may survive the thermal treatment processes (e.g., pasteurization or freezing) or may be introduced through contaminated packaging materials and biofilm proliferation in dead zones where product accumulates, or sanitary practices are insufficient [97,98]. Another significant hazard is the growth of coliform or plate count beyond acceptable levels owing to improper processing conditions or noncompliance with sanitary prerequisites [10,87]. The implementation of NLP; high-speed computer vision to inspect packaging materials; digital twins powered by Ansys Fluent to assess dead zones inside the equipment; and IoT sensors to control humidity, temperature, particulate matter, and ammonia represents a strategy that can help reduce the bacterial load to acceptable levels and support compliance with international food safety guidelines [5,88,99,100].

**Table 2:** Hazard analysis for an ice cream processing line with AI integration.

Process	Type	Hazards	Is This a Probable Hazard?	Hazard Significance	AI Control Measures
Raw material reception	Biological	Pathogenic bacteria (*Listeria*, *Salmonella*, *S. aureus*, and *E. coli*) or elevated coliform/aerobic plate count [101]	Yes	Microbial proliferation due to temperature fluctuations or insufficient thermal treatment, which increases the risk of foodborne hazards [101,102].	IoT temperature sensors linked to cloud data services for cold-chain monitoring in transport vessels [48]. NLP cross-referencing of ingredient specifications against local regulations [31] Decision tree algorithms for historical temperature fluctuation analysis [103]
	Chemical	Milk or raw material adulterants/antibiotics [104]	Yes	Failure to satisfy compositional standards and Certificates of Analysis (COAs) that do not accurately reflect actual batch composition, which could result in a noncompliant product being released into the market [105].	FT-IR/NMR spectroscopic sensors connected to cloud services for real-time physicochemical monitoring and adulteration detection [106]. DL-based classification of raw materials through continuous spectral analysis [107].
	Physical	Physical contaminants (shards of metal, screws, plastic, glass, and environmental debris)	Yes	Contamination originating from milk processing machinery, transport trucks, or inadequate filtration processes compromises the product and consumer safety.	Predictive ML and NLP architectures for high-risk supplier profiling and database management [108,109]. Hyperspectral imaging and computer vision for real-time inspection of incoming materials [40].
Mixing	Biological	Biofilm formation or pathogenic bacteria (*Listeria*, *Salmonella*, *S. aureus*, and *E. coli*).	Yes	The risk of pathogenic survival during mixing, which can be carried into the final product.	Digital twins (Ansys Fluent) for mixing dynamics modeling and biofilm-prone zone identification [110].
	Chemical	Residual detergents, cleaning solutions, allergens, or cross-contamination from prior batches	Yes	Demanding operation schedules, which increase the risk of chemical cross-contamination of the product.	IoT viscometers and FT-IR/NMR spectrometers for real-time viscosity and compositional deviation detection [111].
	Physical	Physical contaminants (e.g., shards of metals and screws)	Yes	Fallen machine parts caused by equipment aging; contamination during transport between stages increases risk of consumer injury.	Computer vision with ML for foreign object detection. IoT sensors for RPM and motor load monitoring [71].
Pasteurization	Biological	Survival of pathogenic bacteria (*Listeria*, *Salmonella*, *S. aureus*, and *E. coli*) [106]	Yes	Inadequate thermal treatment promotes the proliferation of pathogens associated with foodborne illnesses. High temperatures minimize formation of biofilms.	Digital twins (Ansys Fluent) for pasteurizer thermal profiling coupled with IoT temperature sensors and AI-controlled FDVs [112].
	Chemical	Chemical residues from detergents, cleaning solutions, allergens, or prior-batch materials	Yes	Demanding production times and associated structural fractures between the raw material reception and pasteurizing stages cause cross-contamination.	Acoustic sensors for mechanical anomaly detection [113]. CFD modeling within the digital twin environment for chemical residue dead-zone identification [114].
	Physical	Physical contaminants (e.g., metal shards, screws, and gasket remains).	Yes	Equipment wear leads to detachment of machine parts or gasket seal deterioration, increasing the risk of consumer injury.	IoT acoustic and vibration sensors for monitoring the structural integrity of heat exchangers and integration with DL for predictive maintenance [113].
Homogenization and aging	Biological	Biofilm development or pathogenic proliferation	Yes	Failure to enforce strict environmental control increases the risk of pathogen proliferation, which may lead to foodborne illnesses.	IoT sensors for temperature, humidity, and ammonia gas monitoring. Digital twins for dead zone evaluation [115].
	Chemical	Residual detergents, cleaning solutions, mechanical oils, or allergen cross-contamination.	Yes	Demanding schedules increase the risk of residual agents between batches, leading to cross-contamination issues.	Computer vision for homogenizer contamination assessment, cross-referenced with digital twin models [116].
	Physical	Foreign materials (e.g., homogenizer components, hair, and nails)	Yes	Equipment aging causes structural fatigue and metal shard detachment. Manual handling introduces additional contaminants, which pose a risk to consumer safety.	Coupled acoustic sensors and computer vision architecture for predictive maintenance protocol generation [117].
Continuous freezing	Biological	Pathogenic bacteria introduction at the freezer air inlet (*Listeria monocytogenes*)	Yes	Cryogenic operating conditions allow psychrotrophic pathogens such as *L. monocytogenes* to survive low temperatures, increasing the risk of foodborne illnesses.	IoT sensors for particulate matter and pressure differential monitoring with ML algorithms. Digital twins for freezing cylinder flow simulation [75].
	Chemical	Allergen agents or residual substances	Yes	Insufficient changeover protocols or mechanical malfunctions increase the risk of residual cleaning agent carryover, allergenic cross-contamination, and refrigerant leaks.	IoT refrigerant sensors for leak detection. In-line spectrometers for real-time mix composition monitoring [78].
	Physical	Metal shards or blade fragments from scrapers	Yes	Continuous operation induces frictional stress between scraper blades and the freezing cylinder wall, increasing the risk of metal shard contamination.	Acoustic sensors with ML for scraper blade wear detection. Metal detectors for foreign object identification in high-viscosity streams [117,118].
Packaging	Biological	Pathogenic bacteria (*Listeria monocytogenes*) from packaging materials or compromised seals	Yes	High microbial loads from improper storage or contaminated food contact surfaces increase the risk of contamination. Suboptimal temperatures favor the growth of *L. monocytogenes* and associated diseases.	High-speed computer vision for packaging material handling control [119]. Hyperspectral imaging and IoT sensors for microbial load deviation detection [119].
	Chemical	Chemicals from printing inks and adhesives	Yes	High-demand schedules and improper sanitary protocols increase the risk of residual inks, adhesives, or allergen cross-contact.	Hyperspectral imaging for chemical migration and sanitation cross-contamination detection. [120]. NLP for consumer and retailer review analysis.
	Physical	Physical contaminants (e.g., metal shards, mechanical components, and packaging fragments).	Yes	Equipment aging and compromised packaging structure increase the probability of foreign material contamination, which could lead to consumer injury.	Computer vision and hyperspectral imaging for packaging integrity identification [121]. Metal detectors and NLP for noncompliance tracking.
Hardening and storage	Biological	Pathogen survival (specifically *Listeria monocytogenes*) and pest contamination.	Yes	Temperature deviations during storage enable psychrotrophic survival. Inadequate pest control increases contamination risks.	IoT sensors for temperature and humidity monitoring. [122]. Digital twins (Ansys Fluent) for airflow simulation, along with ML for FIFO protocol efficiency assessment [123].
	Chemical	Chemical contaminants (cleaning agents, refrigerant, and sanitizers)	Yes	High-capacity storage operations increase the risk of residual chemical presence, resulting in cross-contamination of the product.	Computer vision for storage facility surveillance. IoT ammonia gas sensors for refrigerant leak detection [124].
	Physical	Physical contaminants from manual handling and residual materials	Yes	Intensive manual handling required during chamber transition increases foreign contaminant risk.	RFID tags for handling automation. Computer vision, metal detectors, and X-ray sensors for detecting foreign objects in the final product [125,126].

#### 4.2.2. Determination of CCPs

**Figure 8:**
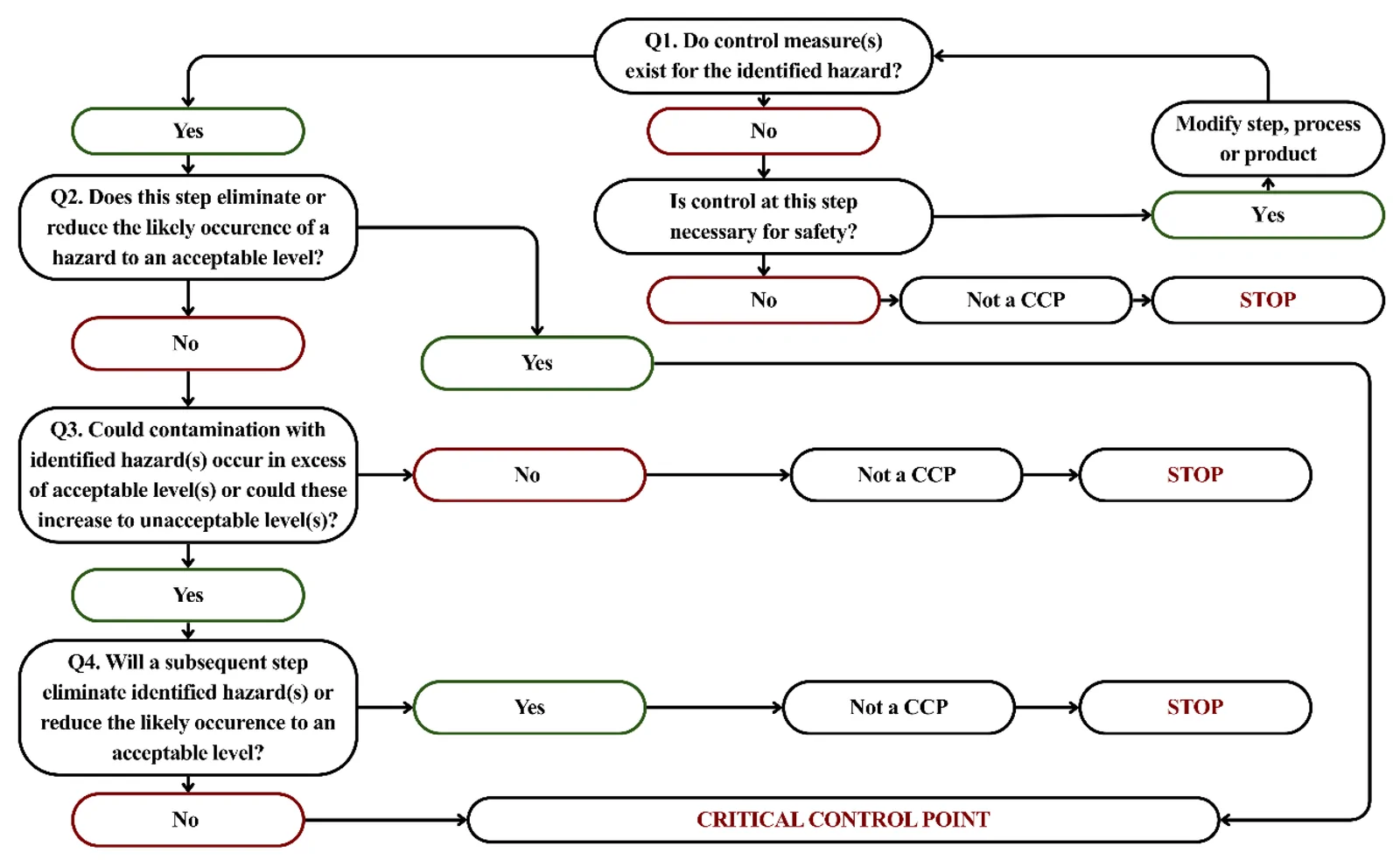
Decision tree established by the USDA Guidelines, which was used for CCP determination. Green boxes indicate an affirmative (“Yes”) response at each decision point; red boxes indicate a negative (“No”) response or a “STOP” outcome (not a CCP).

The determination of CCPs was assessed according to the decision tree established by the USDA regulatory guidelines [38]. This decision tree identifies which unit operations require strict control due to the likelihood of hazard recurrence or the absence of a subsequent step that reduces or eliminates the associated hazard to an acceptable level. Figure 8 illustrates the decision tree applied for CCP determination. The following sequential criteria were evaluated for each unit operation and its associated hazards. The first criterion assesses whether control measures exist for the identified hazard and whether these measures are necessary for product safety. If the assessment is negative, the unit operation is not classified as a CCP. However, modifications to this step, process, or product should be evaluated to determine how control measures can be implemented.

If the first criterion is satisfied, the unit operation is then evaluated to determine whether the process step eliminates or reduces the likelihood of hazard occurrence to an acceptable level. A positive response at this stage classifies the unit operation as a CCP. If the response is negative, a third evaluation is performed to assess whether contamination could occur or increase to unacceptable levels. A negative response at this stage results in a non-CCP classification. If contamination beyond acceptable levels is considered possible, a final assessment determines whether a subsequent step will eliminate the identified hazards or reduce their likelihood of occurrence to an acceptable level. If such a subsequent step exists, the unit operation is not classified as a CCP. If no such step exists, it is designated as a CCP.

Based on these definitions, the CCPs were determined, as shown in Table S1 (Supplementary Materials). This step identified crucial stages such as raw material reception, pasteurization, freezing, and hardening and storage. During these stages, the product is highly prone to bacterial, chemical, or physical contamination, making parameter control crucial to maintain the critical limits established by international regulatory frameworks. Other stages, such as mixing, homogenization and aging, and packaging, were determined to be non-CCPs because, although the hazard assessment identified potential risks during these stages, these risks are effectively managed through a robust prerequisite framework, including sanitary protocols and scheduled inspections, alongside the AI technologies described throughout this review.

#### 4.2.3. Determination of Critical Limits and Monitoring Procedures

It should be clarified that the AI/ML models discussed in this section (including NLP, SVM, and multiple linear regression-based models) are intended to monitor process parameters, predict deviation trends, and support operational optimization around the existing regulatory and industry critical limits summarized above, rather than to establish new critical limits, which remain a regulatory and scientific determination outside the scope of this review. Considering this, critical limits and monitoring procedures were established for the identified CCPs, with a focus on operational parameters, such as temperature, time, air pressure, air speed, and microbial indicators, in compliance with international food safety guidelines. These critical limits and monitoring procedures are summarized in Table 3. For raw material reception, milk and cream must be maintained below 7 °C during transport, and the pH must be between 6.5 and 6.7. Furthermore, the absence of pathogenic bacteria (such as *Listeria monocytogenes*, *Salmonella*, *E. coli*) and adulterants (such as urea, detergents, oils, starch, hydrogen peroxide, and antibiotics) must be confirmed [10,127]. For sugar and flavor additives, the transport temperature must remain below 25 °C; the moisture content must be below 0.06%; microbial parameters such as total plate count (≤100 cfu/g), coliforms (≤30 cfu/g), molds (≤25 cfu/g), and yeasts (≤10 cfu/g) must be strictly controlled within locally established regulatory limits; and the complete absence of pathogenic bacteria must be verified throughout [128]. All parameters must be monitored to prevent recalled batches and microbial proliferation beyond acceptable limits. To implement effective control measures, temperature and humidity sensors must be installed within transport vessels to minimize the probability of early spoilage [8]. FT-IR and NMR spectrometers coupled with computer vision protocols should be employed to enable real-time detection of adulterants or deviations from the critical limits, as they generate automated compliance reports on raw material state [91]. ML and NLP models complement this infrastructure by compiling supplier risk profiles, improving raw material traceability, and reducing the probability of future recalls [3]. All these analyses should be performed each time a new batch of raw materials is received or whenever associated supplier modifications occur. To ensure complete compliance of the monitoring processes, the quality assurance analysts and the supply-chain employees who have reception-designated responsibilities must verify all the procedures.

For pasteurization, the operation temperature must be maintained at 80 °C for 25 s, corresponding to the high-temperature short-time conditions typically applied to frozen dairy dessert mixes. These conditions are crucial since they are CCPs that reduce the risk of microbial proliferation and the presence of pathogens such as *Listeria* monocytogenes [127]. The implementation of temperature and time sensors effectively addresses this and enables AI model training to support compliance with the established critical limits, predictions, and corrective action protocol deployment. The use of digital twins powered by Ansys Fluent optimizes the determination of the heat-transfer flow rate inside the equipment, identifying possible dead zones that might lead to proliferation or survival of pathogens [100]. To ensure proper operation of the pasteurizer, acoustic sensors must be installed to avoid malfunctions and further contamination by chemical or physical agents [129]. Throughout this stage and subsequent operations, analysis must be performed each time a batch is produced. The batch should be assessed by quality assurance analysts and employees responsible for managing the pasteurization process and the remaining CCPs.

During continuous freezing, the temperature must be maintained between −5 °C and −7 °C for approximately 5–6 min [130]. To optimize the ice cream overrun values, the air pressure must be maintained at atmospheric levels, and the injection speed must be fixed at 10–15 m/s [131,132]. The control of these parameters is crucial because the formation of ice crystals with a significant diameter can lead to recalled batches and modifications in their melting behavior, which could result in bacterial survival or proliferation in some cases [133]. IoT temperature and time sensors can be employed to address these challenges, and air-controlled conditions should be monitored to optimize quality parameters while ensuring that injected air does not contaminate the final product. Similar to the case of the pasteurization stage, digital twins can be implemented in this stage to identify thermal dead zones where product safety might be compromised. Additionally, NLP and ML can analyze consumer and retailer reports to support the execution of proper corrective action protocols and automated alerts when there is a deviation from the previously established critical limits.

The final CCP, namely, the hardening and storage stage, is crucial to ensure that the product complies with the established safety parameters and that early spoilage or proliferation of pathogenic bacteria is prevented. Another significant hazard during this stage is cross-contamination between batches beyond the established limits. Considering this, the hardening temperature should be maintained between −40 °C and −18 °C for approximately 20–30 min [134,135,136,137]. During storage, all batches should be chilled at −18 °C to −11 °C, following the FIFO layover supported by ML models [137]. To ensure that these limits are strictly monitored, IoT sensors and the temperature distribution profile created by digital twin models should be applied to identify the possible dead zones where product safety can be significantly compromised within the storage chambers. Computer vision and cameras are used to assess the state of the storage chambers and identify noncompliances associated with refrigerant leaks or cross-contamination hazards.

**Table 3:** Critical limits and monitoring procedures for the CCPs.

Step	Critical Limits		Monitoring Procedures			AI Strategies for Optimization
**What?**	**How?**	**Frequency**	**Who?**			
Raw material Reception	Milk/cream Transport temperature: ≤7 °C [128] pH: 6.5–6.7 [127] Absence of pathogenic bacteria such as *Salmonella*, *Shigella*, *Staphylococcus aureus*, and *Hemolytic streptococcus* [138,139] Absence of adulterants such as urea, detergents, oils, starch, hydrogen peroxide, and antibiotics Sugar/flavor additives transport temperature: ≤25 °C Microbial parameters Total plate count: ≤100 cfu/g Coliforms: ≤30 MPN/100 g Molds: ≤25 cfu/g Yeasts: ≤10 cfu/g Moisture: <0.06%	The transport conditions of the raw materials	Sampling using sensors and spectrometers	Per product lot	QA analysts and supply chain staff	Temperature and humidity sensors for transport condition monitoring In-line spectrometers and computer vision cameras/ML models for contamination detection and traceability. NLP for supplier risk analysis.
Pasteurization	Temperature: 80 °C [136] Time: 25 s [136]	Temperature and time of the pasteurization to reduce the risk of *Listeria monocytogenes*	Use temperature and time sensors that collect data for AI model training	Per batch	QA analysts and production staff	AI models that use sensor data for training in deviation detection. Digital twins (Ansys Fluent) that simulate heat transfer to identify thermal dead zones. Acoustic sensors for verification of equipment operation.
Continuous freezing	Temperature: −5 °C to −7 °C [131] Time: 5–6 min [131] Atmospheric pressure [131] Air speed: 10–15 m/s [132]	Temperature and time are monitored to control ice crystal size and overrun.	Employ temperature/time sensors to prevent pathogen proliferation. Monitor air-controlled conditions to optimize quality parameters.	Per batch	QA analysts and production staff	IoT sensors (temperature, time, pressure, flow) for pathogen control. Digital twins for identification of dead zones NLP/ML models that analyze consumer and operational reports with automated deviation alerts
Hardening and storage	Hardening temperature: −40 °C to −18 °C [135,136] Time: 20–30 min [136,137] Storage temperature: −18 °C to −11 °C [136,137]	Temperature and time are controlled to prevent the proliferation of *Listeria monocytogenes.*	Utilize sensors and computer vision to provide in-depth analysis	Per batch	QA analysts and production staff	IoT sensors that minimize pathogen risk. Digital twins that create temperature distribution profiles for layout optimization. ML models for maintaining FIFO protocol compliance. Computer vision for assessing storage chamber conditions and noncompliances.

#### 4.2.4. Corrective Actions

The corrective actions are essential for efficiently responding to deviations in critical limits, thereby reducing the recurrence of recalled batches or preventing hazards from exceeding the acceptable levels. The corrective action protocols and corresponding AI optimization strategies for each CCP are summarized in Table 4. During raw material reception, all the batches must be identified via RFID/NFC tagging to enable the identification of potentially affected lots of the raw material [94,140]. In the event of a deviation within the specified critical limits, the AI architecture deploys a flow diversion protocol for the unloading operations [87]. Subsequently, the NLP-integrated architecture generates an automatic noncompliance report to the supplier [3]. If there are frequent instances wherein noncompliances exist, ML models compile a risk profile assessment, triggering an alert to transition to another supplier [4]. The corrective actions for the pasteurization stage focus on ensuring that the time and temperature conditions are properly monitored. For this purpose, if a deviation occurs, the FDV redirects the flow for reprocessing or isolating the raw material [141]. Additionally, if there is an operational fluctuation in the equipment, the AI architecture automatically adjusts the steam or pump speed to the established optimal process conditions [100].

Beyond supporting corrective actions, the AI-assisted monitoring framework described throughout Section 4.2 strengthens the preventive philosophy of HACCP by enabling continuous quality assurance and early risk detection. Through real-time analysis of sensor data combined with statistical process control and trend or drift detection, the system can identify process parameters that are gradually approaching critical limits before a deviation occurs. This enables operators to implement timely preventive actions, such as process adjustments, equipment inspection, or additional quality verification, thereby minimizing quality deterioration and reducing the likelihood of food safety incidents. Consequently, the proposed AI-assisted approach shifts HACCP from a predominantly reactive system toward a proactive quality assurance strategy that reduces reprocessing, product recalls, and associated economic losses.

During continuous freezing, the monitoring of time and temperature is relevant to control overall product safety [88]. If there is an anomaly, ML-coupled models, such as HOT-AI, assess the severity of the event by correlating the data from redundant thermal probes and air sensors [64]. For minor fluctuations, the system automatically modulates the refrigerant and air injection flow. However, for major deviations, the system triggers the FDV in the extruder nozzle for further product reprocessing or isolation [87]. Similarly, during hardening and storage, ML models assess the severity of the anomaly by analyzing the temperature, humidity, and ammonia gas data measured by the IoT sensors and then trigger a mandatory quality assessment through computer vision and hyperspectral imaging to inspect the product state [88,93]. Furthermore, if the batch is noncompliant with the established parameters, it should be located using RFID/NFC for rerouting to optimal storage areas, or it should be isolated for final disposition [94]. Digital twin models can identify thermal dead zones in the storage chambers, while ML models can detect deviations from FIFO or routing protocols [4,100].

**Table 4:** Corrective action protocols and AI optimization strategies for CCPs.

Process Step	Corrective Actions	AI Strategies for Optimization
Raw material reception	Affected lot is digitally isolated via RFID/NFC tagging to prevent unauthorized modification. Supplier information is logged for risk profiling,	NFC/RFID identification (blockchain/Gaia-X frameworks) ML-driven FDV approach to halt unloading NLP and ML models for supplier risk profile assessment
Pasteurization	Severity-based dual protocol: If the thermal parameters drop below the critical limit (e.g., <80 °C), FDV activation occurs for re-pasteurization and hazard isolation. Fluctuations within the safety threshold trigger automatic steam flow/pump speed adjustments.	FDV is automatically triggered by the AI architecture in case of a severe anomaly. AI optimization to change the processing conditions (steam flow or flow speed)
Continuous freezing	Severity is assessed via redundant thermal/air sensor correlation. For minor fluctuations, the system triggers refrigerant flow and air injection modulation. For significant safety risks, the system activates FDV at the extruder nozzle for recirculation or isolation.	ML-based severity assessment using HOT-AI FDV is positioned at the extruder nozzle to prevent recontamination. Low-latency hardware/network for rapid decision-making.
Hardening and storage	IoT sensor data (temperature, humidity, and ammonia gas) are assessed for critical limit breaches. The system triggers computer vision/hyperspectral inspection, with noncompliant batches isolated. Digital twins detect dead zones, or FIFO deviation triggers RFID-based batch rerouting to optimal storage.	ML severity assessment IoT sensor network Computer vision/hyperspectral imaging for quality verification Digital twin for dead zone detection RFID-based automatic rerouting

#### 4.2.5. Verification, Validation, and Documentation

For the verification, validation, and documentation protocols, AI and cybersecurity strategies, such as blockchain and XAI protocols, are employed to ensure information safety while reducing the risk of unauthorized modifications and improving the overall traceability throughout the process, as summarized in Table 5 [12,142]. Accordingly, all AI models should be fine-tuned using the SHAP protocol monthly to ensure that the predictions and assessments properly reflect the real-time conditions of the processing line [95]. These records should all be stored in AWS Cloud services [9]. Similarly, the sensors must be calibrated, and the logs related to the audits, quality assessments, critical limit deviation, and the associated deployment of the corrective action must be safely stored [143]. During raw material reception, all lot information, supplier information, and associated RFID/NFC identification data must be documented in the records [94]. Before AI models are deployed for process monitoring or decision support, data collected from different stages of ice cream manufacturing should undergo rigorous validation and quality assessment. Data preprocessing, including cleaning, normalization, and consistency checks, should be performed to eliminate erroneous or incomplete records. Subsequently, datasets should be partitioned into training, validation, and independent test sets, with cross-validation employed to evaluate model robustness. Model performance should also be verified against reference laboratory measurements and established quality control procedures before implementation in industrial HACCP systems to ensure reliable and generalizable predictions.

Where AI models are updated continuously, it is important that this does not translate into fully autonomous compliance classification: any updated model should be re-validated against confirmatory reference-method data before being deployed for CCP monitoring, compliance decisions should retain human-verification checkpoint rather than being issued automatically, and each model version should be documented (training data, performance metrics, deployment date) to preserve auditability, consistent with the screening versus confirmation distinction between the AI/sensor-based methods summarized in Table 3 and standard laboratory reference methods.

For pasteurization and continuous freezing, all condition parameters must be verified each time a new lot is produced, and the sensor-collected data must be safely stored in cloud-based data storage services [9,143]. The fine-tuning and maintenance protocols must be scheduled once a month or according to the volume of production. For this purpose, digital twins can be implemented to create operational reports of the equipment and generate predictive maintenance schedules [100]. Similarly, during hardening and storage, all data must be collected and properly stored. However, in this stage, new employee training protocols and changeover monitoring protocols are crucial to ensure product safety [87]. Specifically, all training records, cleaning protocol logs, and changeover protocol (FIFO) logs are important to improve overall line-level traceability. Technologies such as computer vision, hyperspectral imaging, and digital twins assist in providing a real-time assessment of the state of the storage chamber [87,93,99,100,143]. Furthermore, these technologies allow for the identification of refrigerant leaks, create noncompliance reports, and compile a predictive maintenance schedule [9,100].

**Table 5:** Verification, validation, and documentation procedures for the HACCP plan with AI optimization.

Process Step	Verification Procedures (What, How, Who, and Frequency)	Records	AI Strategies for Optimization
Raw material reception	Parameters checked per lot; monthly sensor/spectrometer calibration; new suppliers undergo risk profile assessment	RFID/NFC lot data, sensor, calibration, QA audits, and deviation/corrective action logs	Blockchain and XAI for data integrity SHAP for model fine-tuning NLP for supplier risk assessment
Pasteurization	Temperature/time logged per batch and uploaded to the cloud; fine-tuning of AI models monthly or upon deviation; monthly maintenance	Temperature/time data; SHAP calibration; maintenance schedules; digital twin-based predictive maintenance reports; deviation/corrective action logs; QA audits	Blockchain and XAI for data security SHAP for fine-tuning Digital twins for predictive maintenance scheduling
Continuous freezing	Parameters checked per lot with blockchain/XAI logging; fine-tuning of AI models monthly or upon critical limit deviation; monthly maintenance	Temperature/time data SHAP calibration Maintenance schedules	Blockchain and XAI for data security SHAP for fine-tuning Digital twins for predictive maintenance
Hardening and storage	Parameters checked per lot with Blockchain/XAI logging; fine-tuning of AI models monthly or upon deviation; monthly maintenance; new employee training on changeover/hygiene protocols	Temperature, time, and ammonia gas data SHAP calibration Maintenance schedules Noncompliance records FIFO logs Sanitary protocol records Digital twin-created dead-zone reports Training records	Blockchain and XAI for data security SHAP for fine-tuning Digital twins for predictive maintenance Computer vision and hyperspectral imaging for storage chamber monitoring

#### 4.2.6. System Implementation and Integration

The implementation of AI technologies within the ice cream processing line offers valuable advantages to improve the HACCP plan development and effectively reduce or eliminate food-safety-associated risks. However, establishing a robust digital architecture is essential to ensure optimal information transmission and data storage in order to minimize the risk of unauthorized modification. As described in the HACCP plan, the transmission of values measured by IoT sensors is crucial for ensuring compliance with the critical limits and for training predictive, classification, and regression ML models. To achieve this objective, the combined implementation of Wi-Fi and LoRaWAN allows for seamless transmission of data across the processing line. This dual-protocol setup ensures connectivity even during service disruptions [62]. All data are stored using cloud services such as AWS or Google Cloud to enhance process traceability [52]. The acquired data are coupled with safety frameworks, including blockchain-based systems for immutability and Gaia-X for secure data sharing, alongside XAI to ensure transparency during external audits [37,52,53,60]. Regular calibration and fine-tuning of the AI models implemented across the processing line are required to avoid false positives. Accordingly, Monte Carlo dropout and SHAP protocols are implemented to ensure predictions reflect real-time operating conditions [64]. Finally, technologies such as MIDAS (Modular ice cream factory dataset) on anomalies in sensors can improve the overall integrability of the unit operations, ensuring that no information gaps compromise the safety of the final product [54].

From an industrial perspective, the proposed framework can be integrated into existing sensor networks and production infrastructure, enabling manufacturers to enhance HACCP performance without fully replacing current quality management systems. AI technologies should complement rather than replace existing quality control (QC) laboratories in ice cream manufacturing. AI-assisted tools, including FT-IR/NMR screening, computer vision, and machine learning models, can provide rapid process monitoring and identify suspect batches for further investigation, while QC laboratories remain responsible for confirmatory microbiological and chemical analyses, instrument calibration, and validation of AI-assisted decisions.

## 5. Future Perspectives and Challenges

The implementation of AI technologies in ice cream processing is effective for assessing food safety hazards and improving traceability, but several challenges remain that will determine the pace of industrial-scale adoption. Economic barriers, particularly the high cost of IoT sensors and digital infrastructure, remain a primary concern for small and medium enterprises (SMEs), though targeted, cost-effective hardware (e.g., single-board computing platforms such as Raspberry Pi) and collaborative data-sharing frameworks such as Gaia-X are lowering this threshold [51,52,60]. A further consideration for industrial adoption is that large-scale ice cream manufacturers already operate highly automated lines with continuous monitoring of temperature, pH, and processing time. In this context, AI-assisted technologies are more realistically introduced as an analytics layer added on top of existing data streams and sensors, rather than as a complete replacement for process equipment, thereby lowering the capital investment for adoption. Because these systems increasingly rely on continuous data acquisition, system reliability and cybersecurity become important considerations. Accordingly, proposed implementations should include redundancy and fail-safe defaults to conventional monitoring in the event of system or connectivity failure, together with routine security testing of connected devices.

Data confidentiality is a related concern, since manufacturers generally treat product formulations and recipes as proprietary intellectual property and may be reluctant to adopt AI systems perceived as increasing the risk of data leakage or cybersecurity breaches. Practical mitigation strategies include encrypted data transmission using Secure Sockets Layer/Transport Layer Security (SSL/TLS) protocols, role-based access control, secure cloud architectures, and edge processing of highly sensitive formulation data, ensuring that confidential manufacturing information remains protected during data transfer and storage.

The adoption of AI-assisted HACCP systems also has important implications for the food manufacturing workforce, particularly in labor-intensive industries in countries such as India and Indonesia, where many processing operations still rely on skilled human operators. Rather than replacing the workforce, AI should be viewed as a decision-support technology that enhances operator performance through real-time monitoring, predictive analytics, and early hazard detection. Successful implementation will require workforce reskilling, continuous training in digital technologies, and the development of human-AI collaborative workflows in which operators retain responsibility for supervision, verification, and critical decision-making. A phased implementation strategy that gradually integrates AI into existing production systems can facilitate workforce adaptation while maximizing both food safety and operational efficiency.

The continuous, high-resolution data generated by hyperspectral imaging and other digital acquisition systems also raise questions of long-term storage: tiered data-management strategies, such as edge preprocessing that retains only exception events or reduced-resolution summaries, together with shared or organization-based cloud infrastructure, may improve the economic feasibility of these systems for SMEs, though this remains an open question for further studies.

Beyond cost and workforce considerations, the regulatory framework for AI in food systems remains fragmented. The European Union’s AI Act classifies high-risk AI systems, imposing conformity and transparency obligations that may apply to autonomous corrective action systems in food processing, while FDA’s current AI framework is oriented toward software as a medical device, leaving a notable regulatory gap for AI-assisted HACCP plans [144]. An AI–HACCP architecture validated under one regulatory framework may require reconfiguration for another, increasing the difficulty for cross-border deployment and integration. This underscores the need for a harmonized international standard governing AI in food safety management.

Data standardization is a related challenge, since differences in equipment calibration, processing protocols, raw material characteristics, and environmental conditions across facilities generate distinct data distributions that can compromise model reliability. Frameworks emphasizing findability, accessibility, interoperability, and reusability (FAIR), together with secure data-sharing architectures such as Gaia-X, provide a structural foundation for addressing cross-facility dataset harmonization while preserving data sovereignty [52,60].

Model interpretability remains a major barrier. Because AI-driven decisions in this context directly affect public health, their operation must extend beyond a “black box” paradigm. Complementary tools such as SHAP or Monte Carlo dropout, combined with XAI architectures that convert model outputs into auditable logs, and continued human oversight of CCP decision-making, are important for regulatory compliance and audit readiness [37,53,64]. Finally, a paradigm shift is warranted in how the HACCP framework itself implements AI: rather than restricting AI-driven control to CCPs alone, it could be implemented as a prerequisite program (PRP) applied across all unit operations, including those that are classified as non-CCPs, where meaningful hazards can still arise but are not currently subject to the same level of control. Accordingly, regulatory bodies, such as the FDA and USDA, must update frameworks, including the *Codex Alimentarius*, to formally integrate AI technologies as PRPs. This regulatory recognition would strengthen process reliability and ensure product safety, without requiring every unit operation to meet the resource-intensive standards implemented for CCPs.

## 6. Conclusions

This review demonstrates that the integration of cutting-edge AI technologies and ML models into HACCP systems represents a paradigm shift for ice cream manufacturing. By establishing a multilayered architecture incorporating IoT sensors, computer vision, and NLP, the system achieves continuous, real-time data collection across the entire processing line. Through HACCP analysis of unit operations, from raw material reception to hardening and storage, key AI strategies were identified to effectively mitigate the associated biological, chemical, and physical hazards. The review successfully identifies four CCPs by applying a critical-limit framework designed to train predictive and classification models capable of executing the established corrective action protocol. Another important aspect of this review is the integration of the HACCP system with cloud services (AWS or Google Cloud) and the implementation of technologies such as blockchain, Gaia-X, or XAI to improve the transparency of the collected data while reducing unauthorized modification risks or possible noncompliant reports from external audits. Although challenges, such as high investment thresholds and the need for human-centric training, persist, the emergence of targeted models and calibration protocols such as SHAP and Monte Carlo dropout provides a pathway toward reliable industrial adoption. Ultimately, the implementation of AI in food safety frameworks represents a robust strategy that extends beyond individual unit operations, serving as a PRP that optimizes the general operations of the processing line.

## Data Availability

No new data were created or analyzed in this study. Data sharing is not applicable to this article.

## Supplementary Materials

The following supporting information can be downloaded at: https://www.mdpi.com/article/10.3390/foods15162815/s1, Table S1. Determination of CCPs using the USDA decision tree.

## Abbreviations

The following abbreviations are used in this manuscript:

AI	Artificial intelligence
AWS	Amazon Web Services
CNN	Convolutional neural network
CCP	Critical control point
CL	Critical limit
CP	Critical point
FDV	Flow diversion valve
FIFO	First-in-first-out
GAN	Generative adversarial network
HACCP	Hazard analysis and critical control point
HOT-AI	Hard-O-Tronic AI
IoT	Internet of Things
LoD	Limit of detection
LoRaWAN	Long-range wide-area network
MIDAS	Modular ice cream factory dataset on anomalies in sensors
ML	Machine learning
NLP	Natural language processing
NFC	Near-field communication
PRP	Prerequisite program
RFID	Radio frequency identification
SHAP	Shapley additive explanations
SME	Small and medium enterprise
SVM	Support vector machine
XAI	Explainable artificial intelligence
