# Supplementary material for: A Review of Machine Learning and AI Applications in Enhancing HACCP Systems for Ice Cream Manufacturing
Source: Foods. 2026 Aug 12;15(16):2815. doi: 10.3390/foods15162815 (PMC13512804; doi:10.3390/foods15162815)
Supplement: Supplementary file 1 [file foods-15-02815-s001.zip › foods-4481617-supplementary.pdf]

*Supplementary Materials*

The following supporting information is provided: Table S1: Determination of CCPs using the USDA decision tree.

**Table S1.** Determination of CCPs using the USDA decision tree.

| Process Step | Hazard | Q1 | Q2 | Q3 | Q4 | CCP |
|---|---|---|---|---|---|---|
| Raw material reception | Biological | Yes | Yes | NA | NA | Yes |
| | Chemical | Yes | Yes | NA | NA | |
| | Physical | Yes | Yes | NA | NA | |
| Mixing | Biological | Yes | No | No | NA | No |
| | Chemical | Yes | No | No | NA | |
| | Physical | Yes | No | No | NA | |
| Pasteurization | Biological | Yes | Yes | NA | NA | Yes |
| | Chemical | Yes | Yes | NA | NA | |
| | Physical | Yes | Yes | NA | NA | |
| Homogenization and aging | Biological | Yes | No | No | NA | No |
| | Chemical | Yes | No | No | NA | |
| | Physical | Yes | No | No | NA | |
| Continuous freezing | Biological | Yes | No | Yes | No | Yes |
| | Chemical | Yes | No | Yes | No | |
| | Physical | Yes | No | Yes | No | |
| Packaging | Biological | Yes | No | No | NA | No |
| | Chemical | Yes | No | No | NA | |
| | Physical | Yes | No | No | NA | |
| Hardening and storage | Biological | Yes | No | Yes | No | Yes |
| | Chemical | Yes | No | Yes | No | |
| | Physical | Yes | No | Yes | No | |
